# Invasive Amebiasis in Men Who Have Sex with Men, Australia

**DOI:** 10.3201/eid1407.080017

**Published:** 2008-07

**Authors:** Damien Stark, Sebastian J. van Hal, Gail Matthews, John Harkness, Deborah Marriott

**Affiliations:** * St. Vincent's Hospital, Darlinghurst, New South Wales, Australia; † University of New South Wales, Sydney, New South Wales, Australia

**Keywords:** Entamoeba histolytica, amebiasis, men who have sex with men, dispatch

## Abstract

*Entamoeba histolytica* is a pathogenic ameba that has recently been recognized as an emerging pathogen in men who have sex with men (MSM) in Asia-Pacific countries where it is not endemic, i.e., Japan, Taiwan, and Republic of Korea. We report locally acquired invasive amebiasis in Sydney, Australia, exclusively in MSM.

*Entamoeba histolytica* is a pathogenic ameba that can cause invasive intestinal and extra-intestinal disease. The most frequent manifestations of invasive amebiasis are colitis and liver abscesses (*1*). Although *E. histolytica* is one of the most common parasitic infections worldwide, invasive disease remains uncommon in industrialized counties. Recent studies from Japan, Taiwan, and Republic of Korea, areas where *E. histolytica* endemicity is generally low, suggest that amebiasis is an emerging parasitic infection that occurs exclusively in men who have sex with men (MSM) (*2*–*6*). In Australia, the documented incidence of *Entamoeba* spp. in the general population is 1%–4% (*1*). In MSM, the rates of *Entamoeba* spp. carriage were previously documented to be as high as 37% (*7*). However, these studies failed to differentiate the pathogenic *E. histolytica* from the morphologically identical nonpathogenic *E. moshkovskii* and *E. dispar*, therefore leaving the accuracy of these results in question. A study in Australia that used molecular methods showed *E. histolytica* prevalence rates in MSM to be as low as 0.1% (*8*). Impaired host immunity is associated with increased pathogenicity of invasive amebiasis. Recent studies indicate an increased risk for invasive amebiasis among persons with HIV (*9*,*10*). We report 5 cases of invasive amebiasis in MSM from Sydney, New South Wales, Australia, from December 2006 through October 2007. 

## The Cases

Of the 5 patients, 3 had amebic colitis and 2 had amebic liver abscesses, all were MSM, 4 were HIV-infected, mean age was 45 years (range 35–57 years), and median CD4 count was 713 cells/mm^3^ (Table). No associations among any of the patients were noted. All patients were receiving highly active antiretroviral therapy at the time of examination. Of the 5 patients, 4 had not traveled extensively in the past 4 years, but the remaining patient had traveled to Malaysia and China 6 months before onset of colitis. Other possible risk factors for acquisition were identified in 4 patients: 3 had had high-risk sex behavior at sex-on-premises venues, and the other had had a male partner who had traveled to countries where invasive amebiasis was highly endemic.

**Table:** Summary of clinical characteristics of 5 patients with invasive amebiasis, Australia, December 2006–October 2007*

Age, y	HIV status	CD4 count, cells/mm^3^	HIV viral load, copies/mL	Clinical condition	Identified risk factors
35	+	672	ND	Colitis	SOP
42	+	756	<40	Colitis	SOP and travel in past 6 mo
43	+	754	ND	Colitis	SOP
57	–	ND	ND	Liver abscess	Extensive travel by ex-partner in past 8 mo
52	+	256	30,700	Liver abscess	None

*ND, not done; SOP, visited sex-on-premises venue.

The patients had bloody diarrhea and abdominal pain for 2–4 weeks. Routine fecal cultures were negative for bacterial pathogens. Microscopic examination of permanently fixed, stained fecal smears was positive for *E. histolytica*/*dispar/moshkovskii* complex. Diagnosis of *E. histolytica* was confirmed by PCR targeting the small subunit ribosomal DNA as described (*8*). Results of serologic examination of these patients were positive; all titers were >256 according to indirect hemagglutination antibody assay, which confirmed invasive disease. However, although antibodies against amebae indicate invasive disease, these antibodies can also be seen in persons with asymptomatic colonization with amebae (*1*). 

The 2 patients with liver abscesses each had large, solitary abscesses in the right lobe of the liver (8 × 6 × 6 cm and 7.5 × 6.2 × 6.6 cm). In 1 patient, the abscess ruptured through the liver capsule, and collapse of the right middle and lower lung lobes and a resultant pleural effusion complicated the subphrenic collection of pus from the abscess. Levels of liver enzymes (alkaline phosphatase and gamma glutamyl transferase), C-reactive protein, and neutrophils (absolute numbers) were raised. Both patients underwent percutaneous drainage of their liver abscess; cultures of aspirated pus were negative for bacteria and fungi. Results of indirect hemagglutination antibody assay were positive; titer was >256. Microscopy and PCR of fecal samples were negative for *E. histolytica.* For these 2 patients with amebic liver abscess, the diagnosis was delayed; they had had symptoms for >2 weeks and were then treated for bacterial liver abscess before the correct diagnosis was made. 

All 5 patients made a successful recovery after treatment with metronidazole. In addition, all were treated for cyst carriage with paromomycin, a luminal amebicide.

## Conclusions

*E. histolytica* carriage and invasive disease are common in the Asia-Pacific region, especially in developing countries. In countries where *E. histolytica* prevalence is low, such as Japan, Taiwan, Republic of Korea, and Australia, rates of amebiasis are low and invasive amebiasis is uncommon. Recent reports from a number of these countries, however, suggest that invasive amebiasis is emerging as an increasingly common infection, specifically in the MSM population (*2*–*6*). MSM have a higher risk than others for intestinal parasite carriage; not only are they substantially more likely to harbor intestinal protozoa, but they are also more likely to harbor multiple parasites (*11*). These protozoa are transmitted by the fecal–oral route; high rates of oral–anal sex by MSM are considered the reason for increased rates of carriage. Because *E. histolytica* is also transmitted by the fecal–oral route, MSM may also have an increased risk for *E. histolytica* carriage. This higher rate of asymptomatic carriage is likely to translate into a greater risk for invasive disease. Recent seroprevalence studies in Taiwan that used indirect hemagglutination antibody assay have confirmed MSM’s statistically significant higher risk for *E. histolytica* exposure (*5*).

In Japan, amebiasis has become endemic in MSM; symptomatic *E. histolytica* infection occurs almost exclusively in middle-aged MSM in the large cities of Japan (*2*,*3*). Similar findings are reported for MSM in Taiwan (*4*,*5*). More recently, a study from the Republic of Korea documented invasive amebiasis (amebic liver abscess) in HIV-infected MSM (*6*). To date, the emergence of *E. histolytica* infections in MSM seems to be limited to the Asia-Pacific region. In a large retrospective study of 34,000 HIV-infected patients in United States, only 2 patients had invasive amebiasis (*12*). The reasons for this geographic variation are unclear, but it is likely linked to the higher background prevalence of *E. histolytica* infections in Asia. Regional *E. histolytica* strains show a high degree of diversity but no major differences between regional genotypes; other factors relating to host or virulence factors may be important but are as yet undetermined (*3*). We now report local acquisition of *E. histolytica* by MSM in Australia; as shown by the above 5 cases of invasive disease and 3 previous cases of noninvasive infections also acquired locally (*13*).

Of note, 4 of the 5 patients we report were HIV infected. Seroprevalence rates of *E. histolytica* (determined by indirect hemagglutination antibody assay) are higher for HIV-infected persons then for HIV-nonninfected persons, although the reasons are unclear (*5*,*14*). Higher rates of *E. histolytica* carriage in MSM likely reflect high-risk sex behavior and multiple exposures, resulting in increased risk for acquisition. This hypothesis is supported by the high rates of sexually transmitted infections that occur in Australian MSM who visit sex-on-premises venues (*15*). Antibody responses predominantly occur with invasive disease. Whether immunosuppression caused by HIV infection attenuates the risk for invasive amebiasis is unknown. Historically, the evidence has been contradictory, and most published studies had had severe limitations. Nevertheless, more recent data seem to indicate that HIV-infected persons are at increased risk for invasive amebiasis (*9*,*10*).

The emergence of *E. histolytica* in MSM is of public health concern because it has the potential to become endemic in this population in Australia and to cause severe disease. Further study is needed to identify the reasons for the geographic variation and the role of *E. histolytica* in invasive disease. In conclusion, invasive amebiasis has the potential to emerge as an important parasitic infection in the Asia-Pacific region, especially in HIV-infected MSM in countries where *E. histolytica* is not endemic.
